# The Impact of COVID-19 Pandemic on Searching for Health-Related Information and Cyberchondria on the General Population in Italy

**DOI:** 10.3389/fpsyt.2021.754870

**Published:** 2021-10-12

**Authors:** Matteo Vismara, Daniele Vitella, Roberta Biolcati, Federica Ambrosini, Veronica Pirola, Bernardo Dell'Osso, Roberto Truzoli

**Affiliations:** ^1^Department of Biomedical and Clinical Sciences “Luigi Sacco”, University of Milan, Milan, Italy; ^2^“Aldo Ravelli” Center for Neurotechnology and Brain Therapeutic, University of Milan, Milan, Italy; ^3^University of Milan, Milan, Italy; ^4^Department of Education Sciences “Giovanni Maria Bertin”, University of Bologna, Bologna, Italy; ^5^UniSR-Social Lab, Faculty of Psychology, Vita-Salute San Raffaele University, Milan, Italy; ^6^Department of Psychiatry and Behavioral Sciences, Bipolar Disorders Clinic, Stanford University, Stanford, CA, United States; ^7^“Centro per lo studio dei meccanismi molecolari alla base delle patologie neuro-psico-geriatriche”, University of Milan, Milan, Italy

**Keywords:** Cyberchondria, online health searches, COVID-19, mental health, health anxiety, digital addiction

## Abstract

**Objectives:** The Internet has become one of the most common sources people use to search for health-related information, a behavior rapidly increased during the novel Coronavirus disease 2019 (COVID-19). The present study aimed to investigate behavioral patterns in the online health-related searches and Cyberchondria (CYB) during the COVID-19 pandemic time, in order to explore socio-demographic and psychopathological factors related to CYB.

**Methods:** During the third wave of the COVID-19 pandemic in Italy, a cross-sectional online survey collected the main socio-demographic variables and habits related to Internet use of 572 participants. CYB was measured by the Cyberchondria Severity Scale-Short Version and different psychopathological factors were measured by specific questionnaires: the Coronavirus Anxiety Scale, the Hospital Anxiety and Depression Scale, the Short Health Anxiety Inventory, the Meta-Cognitions about Health Questionnaire, the Internet Addiction Test, the Maudsley Obsessional-Compulsive Questionnaire-Short Version, the Rosenberg's Self-Esteem Scale, and the WHO Quality of Life-BREF. Descriptives, non-parametric ANOVAs, and Spearman correlations were performed.

**Results:** In the present sample, the Internet was the main source participants used to search for health-related information and nearly one-third increased this habit during the pandemic. Higher expression of CYB emerged in females, in younger participants, in students, and in those suffering from a physical/psychiatric illness. CYB showed a positive correlation with different phenomenology of anxiety (i.e., anxiety about COVID-19, health anxiety, general anxiety, metacognitive believes about anxiety) and with depression, obsessive-compulsive symptoms, and problematic usage of the Internet. Conversely, quality of life and self-esteem showed a negative correlation with CYB.

**Conclusion:** During the COVID-19 pandemic, the use of the Internet for health-related information and CYB contribute to the psychological stress affecting individuals and society. Delineating subjects more vulnerable to CYB and associated psychopathological factors will help to elaborate operational indications for prevention and psychological support.

## Introduction

Since its outbreak in December 2019, the current Coronavirus (SARS-CoV-2) COVID-19 pandemic has been responsible for high morbidity and mortality rates but also for considerable physical and psychological stress (1–5). In a constantly evolving situation, Italy counts approximately 4.2 million infections (6% of the population) (6) with the epidemic curve picturing thus far three waves that reflect not only an increase/decrease in the number of new infections/hospitalizations/deaths but also a repetitive waxing and waning of psychological stressors for the individuals.

The unprecedented restriction of face-to-face contacts and the requirement to stay at home for prolonged periods of time, combined with financial, health, and occupational worries, caused a relevant impact on mental health (7–9) and influenced people's risky tendency to engage in potentially addictive behaviors (10, 11), including Internet addictions (12, 13). A risky online activity particularly worthy of attention during a public health emergency is the tendency to make excessive online health-related searches (14). To cope with the current crisis, when it is difficult to access face-to-face health professionals counseling, it is quite natural for people to turn more frequently to the Internet in order to find COVID-19 health-related information, for example protective behaviors (15, 16). A study conducted on the Chinese general population found that 93.5% of the sample used the Internet as the most common source of health information about COVID-19. In the same investigation, specific up-to-date and accurate health information (e.g., treatment, local outbreak situation) and particular precautionary measures (e.g., hand hygiene, wearing a mask) were associated with lower psychological impact (5). On the other hand, excessive searching of health information could potentially increase the level of stress, anxiety, and depression, as reported in other recent investigations (17, 18). Considering the huge amount of health-related information available online, an increased risk of fake news and unverified information have been reported in the current pandemic era (particularly favored by the use of social media) (19, 20), with consequent amplification of one's perceived risk, anxiety, and negative impact on well-being and quality of life (20–23).

Yet before the impact of the COVID-19 pandemic, a negative behavior characterized by excessive and repetitive online health research combined with increasing levels of health anxiety or distress has been described and defined as “Cyberchondria” [CYB; (24–26)]. CYB may represent a trans-diagnostic syndrome linked to different classes of disorders, showing a complex nosological relationship with health anxiety (27), problematic usage of the Internet (28), and obsessive-compulsive disorder (25). Additionally, CYB shows various underlying psychological mechanisms likely reinforcing CYB that mostly include transdiagnostic factors related to these disorders, in particular, metacognitive beliefs about health anxiety (29–32) and self-esteem (33).

COVID-19 pandemic reasonably represents a fertile soil for the development of CYB: people are experiencing a heightened perception of threat and fear of a newly identified and poorly understood disease; are at higher risk to be exposed to information overload (with a potentially consequent risk of misinformation); and find higher difficulty in coping with uncertainty and in being reassured by the information found online (34–36). Several researches already described and measured CYB during the COVID-19 outbreak (34, 37–41). Indeed, CYB has been reported as a contributing factor for the “Coronavirus anxiety” (35–37) and seems to be favored by trust and acceptance of online information (22, 42), information overload (22, 40), perceived vulnerability to COVID-19 (40), and by the use of social media as main source of information (43). In a sample of university students investigated during the first part of the COVID-19 outbreak, CYB seems to be predicted by female gender (22, 38), living alone, and by the belief of having contracted the virus (38).

According to literature data, the analysis of behavioral and psychological effects of the virus spread has mainly been focused on the first time lapse of the COVID-19 outbreak. On the contrary, little is still known about the same effects in the longer pandemic time that has seen the introduction of differentiated lockdowns and subsequent relaxation of social distancing measures. So far, it seems that only few studies have been focused on the influence of sociodemographic and, in particular, of COVID-19 related variables on CYB. Moreover, only few literature studies have been focused on the course of the pre-existing psychopathological CYB-related factors (e.g., health-anxiety) during the pandemic time. Considering the negative impact CYB might have either on personal [i.e., escalated anxiety and distress (24, 25)] and societal level [i.e., increase in health care utilization (23)], it seems to be of a relevant significance to deepen the understanding of the role of sociodemographic and psychopathological variables as predisposing and/or maintaining factors of CYB.

Accordingly, this cross-sectional study was aimed, in a large sample of the general Italian population, to (a) describe the behavioral patterns related to online health searches and to CYB; (b) identify sociodemographic and psychopathological factors associated to increased expression of CYB.

## Materials and Methods

### Design and Participants

A cross-sectional survey was conducted among the Italian general population during the third wave of the COVID-19 pandemic (February 1st to April 30th, 2021) by an anonymous online questionnaire. A snowball strategy was adopted. The online survey was firstly spread through University mailing lists and groups on social media platforms, then encouraging these initial participants to pass it on to other acquaintances. Participants gave their informed consent and completed the survey via an online platform (Google Forms, Google LLC, 1600 Amphitheater Parkway, Mountain View, CA 94043, USA). An answer to each question was required to submit the survey and each participant (limiting for personal account) could complete the survey only once. The study was performed in accordance with the ethical standards as laid down in the 1964 Declaration of Helsinki and its later amendments; ethical approval was obtained from the University of Milan Ethics Committee (number 27/21).

### Assessments

The following sociodemographic variables were collected: age, residence area, gender, the highest level of education achieved, occupation, relationship and living status, physical or psychiatric illness, and family history of mental disorders. Additionally, participants were asked about their behavioral patterns in browsing the web especially health-related navigation. Lastly, participants were asked to report if they have tested positive for SARS-CoV-2, if any relative had contacted the virus or had passed away because of COVID-19. The Appendix section reports the detailed list of questions.

In order to measure CYB symptoms severity, the Italian short version of the Cyberchondria Severity Scale [CSS-12, (44)] was administered. This self-reported questionnaire consists of 12 items rated on a 5-point scale from 1 (never) to 5 (always) including the four CYB subscales: compulsion, distress, excessiveness, and reassurance (45). Previous investigations and a recent systematic review on the CSS showed very good to excellent psychometric properties for the CSS (46). In the study sample, the CSS-12 showed a high level of internal consistency (Cronbach's alpha of 0.88).

In light of literature data, the Italian version of the following self-reported validated questionnaires was administered to measure the correlation between psychopathological factors and CYB.

*The Coronavirus Anxiety Scale* [CAS, (47)] was used to assesses dysfunctional anxiety associated with COVID-19. The CAS consists of 5-item rated on a 5-point scale. In the current sample, the CAS has shown a good internal consistency (Cronbach's alpha of 0.84).

*The Short Health Anxiety Inventory* [SHAI, (48)], consisting of 18 items rated on a four-point scale, was used to measure the severity of health anxiety symptoms. In the current sample, the SHAI reported a high internal consistency (Cronbach's alpha of 0.89).

*The Hospital Anxiety and Depression Scale* [HADS, (49)], consisting of 14 items scored from 0 to 3 and divided into two subscales, was used to determine the severity of general anxiety and depressive symptoms. Cronbach's alpha was 0.85 for the anxiety and 0.77 for the depression subscale.

*The Meta-Cognitions about Health Questionnaire* [MCQ-HA, (50)] consists of 14 items rated on a 4-point scale and measures health anxiety-related metacognitions. In the present study, the MCQ-HA showed a good internal consistency (Cronbach's alpha of 0.85).

*The Internet Addiction Test* [IAT, (51)] measures, through 20 items rated on a 5-point Likert scale, the severity of Internet addiction. In the present study, the IAT showed a high internal consistency (Cronbach's alpha of 0.91).

*The Maudsley Obsessional-Compulsive Questionnaire* [MOCQ-Short Version, (52)] is a 21-item with true-false format questionnaire measuring the severity of obsessive-compulsive symptoms. The Cronbach's alpha coefficient of the MOCQ-R was 0.81.

*The Rosenberg Self-Esteem Scale* [RSES, (53)] assesses global self-worth by measuring both positive and negative feelings about the self. The RSES includes 10 items measured on a 4-point Likert scale. In the present study, internal consistency was high (Cronbach's alpha of 0.89).

*The WHO Quality of Life-BREF* [WHOQOL-BREF, (54)] measures participants' perceptions of their health and well-being. The WHOQOL-BREF is an abbreviated version of the WHOQOL-100 tool consisting of 26 items rated on a 5-point Likert scale. Cronbach's alpha was 0.83.

### Statistical Analyses

Statistical analyses were performed using Statistical Package for the Social Sciences (SPSS) version 26 software. First, descriptive analyses were performed in the whole sample. Qualitative variables were described by frequency and percentage and continuous normally distributed variables were described by mean and standard deviation. Skewness and kurtosis indexes and Q-Q plots showed a non-normal distribution of CYB. Therefore, non-parametric models were adopted. Specifically, to analyze the impact of age on CYB, a generalized linear model using the gamma link function was performed. Moreover, a set of Kruskal-Wallis tests were used to analyze the impact of socio-demographic variables on CYB. Dwass-Steel-Critchlow-Fligner test was used for pairwise comparisons. Median and interquartile range (IQR) were used to report the levels of CYB in socio-demographic and clinical variables. Spearman's correlation coefficient was used for analyzing the relationships between continuous variables in the whole sample. For all the analyses, the level of statistical significance was set at 0.05.

## Results

### Sample Description

The overall sample consisted of 572 participants (mean age 33.6 ± 14.6 years; females 65.6%). The survey collected data from different Italian regions, with the most participants living in Lombardy (*n* = 259, 45.3%), Tuscany (*n* = 94, 16.4%), Emilia-Romagna (*n* = 59, 10.3%), or Apulia (*n* = 42, 7.3%). With respect to the clinical variables, most of the subjects reported not suffering of physical or mental illness (*n* = 419, 73.3%); among sufferers, 7.3% (*n* = 42) reported a psychiatric disease.

### Influence of Sociodemographic Variables on Cyberchondria

Table 1 outlines sociodemographic variables and associated scores on the CSS-12.

**Table 1:** Socio-demographic variables showing a statistically significant difference on the Cyberchondria Severity Scale (CSS-12).

**Variables**		**Mean (±SD) – *N* (%)**	**CSS-12 median + IQR (Q1–Q3)**	***P*-value**
Total sample		572	**19.0 (15.0–25.0)**	–
Age^a^		33.6 (±14.6)	–	**0.002**
Gender^b^				**0.028**
	**Male**	**197 (34.4%)**	**18.0 (15.0–24.0)**	
	**Female**	**375 (65.6%)**	**20.0 (16.0–26.0)**	
Education^b^				0.464
	High School	266 (46.5%)	20.0 (15.0–25.75)	
	University degree	120 (21%)	19.0 (15.0–24.0)	
	Post-university degree	33 (5.8%)	19.0 (15.0–26.0)	
Occupation^b^				**0.028**
	Employed	241 (42.1%)	19.0 (15.0–25.0)	
	**Retired**	**35 (6.1%)**	**17.0 (13.0–20.0)**	**Retired vs. student: 0.016**
	**Student**	**264 (46.2%)**	**20.0 (16.0–26.3)**	
	Housewife	13 (2.3%)	18.0 (13.0–26.0)	
	Unemployed	19 (3.3%)	19.0 (17.0–24.5)	
Relationship status^b^				0.152
	Married	120 (21%)	19.0 (15.0–23.25)	
	Single	423 (74%)	20.0 (16.0–26.0)	
	Widower	9 (1.6%)	20.0 (18.0–25.0)	
	Separated	9 (1.6%)	21.0 (18.0–26.0)	
	Divorced	11 (1.9%)	17.0 (14.0–18.5)	
Living status^b^				0.792
	Family of origin	284 (49.7%)	19.5 (16.0–25.0)	
	Alone	120 (21%)	19.0 (15.0–26.0)	
	Acquired family	168 (29.4%)	20.0 (15.0–25.0)	
Any comorbid disease^b^				**0.019**
	**No**	**419 (73.3%)**	19 (16.0–27.0)	
	**Yes**	**153 (26.7%)**	21 (15.0–25.0)	
Psychiatric comorbid disorder^b^				**<0.001**
	**No**	**530 (92.7%)**	19.0 (15.0–25.0)	
	**Yes**	**42 (7.3%)**	25.0 (20.0–31.0)	
Family history of psychiatric disorders^b^				0.134
	No	77 (74.8%)	20.0 (16.0–25.0)	
	Yes	26 (25.2%)	27.0 (15.8–33.0)	

a *ANOVA*.

b *Kruskal-Wallis test. IQR, interquartile range*.

*Bold indicates a significant difference*.

The first relevant data relates to higher CYB symptom severity in female responders compared with males [Kruskal-Wallis test, ${\text{X}}_{\left(1\right)}^{2}$ = 4.84, *p* = 0.028].

Additionally, the CSS-12 scores were significantly different in relation to the age of participants [b = −0.003, SE = 0.0010, Wald ${\text{X}}_{\left(1\right)}^{2}$ = 9.3, *p* = 0.002], with younger subjects showing higher CYB symptom severity.

Moreover, statistically significant differences emerged with respect to the occupation [Kruskal-Wallis test, ${\text{X}}_{\left(4\right)}^{2}$ = 10.8, *p* = 0.028]. Indeed, at Dwass-Steel-Critchlow-Fligner pairwise comparisons, retired participants showed a statistically lower CYB symptom severity (W = 4.409, *p* = 0.016) compared with students. In addition, subjects who reported having any comorbid disease [Kruskal-Wallis test, ${\text{X}}_{\left(1\right)}^{2}$ = 5.5, *p* = 0.019] or suffering from a psychiatric disorder [Kruskal-Wallis test, ${\text{X}}_{\left(1\right)}^{2}$ = 13.3, *p* < 0.001] showed a significantly higher CYB symptom severity. No differences on the CSS-12 scores emerged with respect to the highest level of education (*p* = 0.464), relationship (*p* = 0.152) or living status (*p* = 0.792), and family history of psychiatric disorders (*p* = 0.134).

### Influence of COVID-19 Related Variables on Cyberchondria

Considering variables relative to the experience individuals had with SARS-CoV-2 (Table 2), 11.4% of participants have tested positive and 40.2% have at least one relative who has been infected. The 9.8% reported the loss of a dear one because of COVID-19. No significant difference in CYB symptom severity emerged in relation to having been infected, having a relative who tested positive, or having lost someone because of COVID-19.

**Table 2:** Variables related to COVID-19 and associated scores on the Cyberchondria Severity Scale (CSS-12).

**Variables**		**Total sample *N* (%)**	**CSS-12 median + IQR (Q1–Q3)**	***P*-value^a^**
SARS-CoV-2 infections				0.53
	No	507 (88.6%)	19.0 (16.0–25.0)	
	Yes	65 (11.4%)	20.0 (15.0–28.0)	
SARS-CoV-2 infections between relatives				0.74
	No	342 (59.8%)	19.0 (16.0–25.0)	
	Yes	230 (40.2%)	19.0 (15.0–25.0)	
Loss of a loved one				0.58
	No	516 (90.2%)	19.0 (15.0–26.0)	
	Yes	56 (9.8%)	18.5 (15.8–23.3)	

a *Kruskal-Wallis test*.

### Influence of Online Health Searches on Cyberchondria

Table 3 outlines the results of variables related to online health searches and associated scores on the CSS-12.

**Table 3:** Variables related to online health searches and associated scores on the Cyberchondria Severity Scale (CSS-12).

**Variables**		**Total sample *N* (%)**	**CSS-12 median + IQR (Q1–Q3)**	***P*-value^a^**
Time spent on the Internet^b^				0.05
	<1 h	32 (5.6%)	18.5 (15.5–22.3)	
	1–3 h	203 (35.5%)	18.0 (15.0–−24.0)	
	3–5 h	174 (30.4%)	21.0 (16.0–27.8)	
	>5 h	163 (28.5%)	19.0 (16.0–25.0)	
Increase in online health searches after the COVISD-19 pandemic				**<0.001**
	**Much less than before**	**30 (5.2%)**	**16.5 (12.5–20.0)**	**Much less than before vs. More than before: 0.037^b^**
	**Less than before**	**62 (10.8%)**	**19.0 (15.0–23.8)**	**Less than before vs. Much more than before:** **<** **0.001^b^**
	**As before**	**303 (53%)**	**19.0 (15.0–24.0)**	**As before vs. Much more than before:** **<** **0.001**
	**More than before**	**124 (21.7%)**	**21.0 (17.0–28.0)**	**As before vs. More than before: 0.025^b^**
	**Much more than before**	**53 (9.3%)**	**26.0 (20.0–32.0)**	**Much less than before vs. Much more than before: 0.001** **More than before vs.: Much more than before: 0.037^b^**
Frequency of online health information search related to COVID-19				0.13
	Less than once a week	216 (37.8%)	19.0 (15.0–24.0)	
	Once a week	139 (24.3%)	19.0 (16.0–25.0)	
	Every 3/4 days	126 (22%)	19.0 (15.0–26.0)	
	Every day	84 (14.7%)	22.0 (17.0–26.0)	
	Several times a day	7 (1.2%)	18.0 (13.5–19.5)	
Source used for online health information search				**<0.001**
	**Health professional/general practitioner**	**183 (32%)**	**18.0 (15.0–22.5)**	**Health professional/general practitioner vs. Internet:** **<** **0.001^b^**
	**Internet**	**211 (36.9%)**	**23.0 (18.0–29.0)**	**Internet vs. scientific journals/encyclopedias etc.:** **<** **0.001^b^**
	**Scientific journals/encyclopedias etc**.	**109 (19.1%)**	**19.0 (15.0–24.0)**	
	Other	19 (3.3%)	18.0 (16.0–22.5)	
	TV/radio	34 (5.9%)	19.0 (17.0–24.8)	
	Non-professional information by friends and relatives	16 (2.8%)	17.5 (14.3–22.3)	
To find struggle in stopping to search for COVID-19 related information on the Internet				**<0.001**
	**Never**	**327 (57.2%)**	**18.0 (15.0–23.0)**	
	**Rarely**	**135 (23.6%)**	**21.0 (17.0–28.0)**	**Rarely vs. never:** ** <0.001^b^**
	**Occasionally**	**88 (15.4%)**	**23.0 (17.0–29.0)**	**Occasionally vs. never:** ** <0.001^b^**
	**Frequently**	**18 (3.1%)**	**27.5 (21.5–36.0)**	**Frequently vs. never:** ** <0.001^b^**
	Always	7 (0.7%)	18.5 (15.0–24.0)	
To believe in online information related to COVID-19 without verifying their source and validity				0.1
	Never	385 (67.3%)	19.0 (15.0–24.0)	
	Rarely	105 (18.4%)	20.0 (17.0–26.0)	
	Occasionally	62 (10.8%)	21.0 (17.0–28.75)	
	Frequently	17 (3%)	24.0 (15.0–30.0)	
	Always	3 (0.5%)	21.0 (16.5–31.0)	

a *Kruskal-Wallis test*.

b *Dwass-Steel-Critchlow-Fligner pairwise comparisons*.

*Bold indicates a significant difference*.

During the COVID-19 pandemic time, the Internet was the most used source for health-related information (36.9%), while 32% of the participants reported preferring a health professional/general practitioner counseling or scientific papers/encyclopedias (19.1%). In the whole sample, CYB symptoms severity was different with respect to the source participants used to obtain medical information [Kruskal-Wallis test, ${\text{X}}_{\left(5\right)}^{2}$ = 40.8, *p* < 0.001]. In detail, at Dwass-Steel-Critchlow-Fligner pairwise comparisons, participants who reported to refer to the Internet as the main source of information showed statistically higher CSS-12 total score than individuals referring to a health professional/general practitioner (W = 8.44, *p* < 0.001) or to scientific journals/encyclopedias (W = 5.74, *p* < 0.001). A trend toward significance (*p* = 0.05) emerged with respect to average time spent online (for any reason), with subjects spending more time online reporting higher CSS-12 scores.

Moreover, one in two participants did not change the frequency of surfing for health-related information (53%), while participants who reported an increase in this behavior were numerically higher (31%) than subjects who reported to have reduced it (16%). In this respect, in the total sample, CYB symptoms severity showed a significant difference according to the change in the frequency of online health-related searches [Kruskal-Wallis test, ${\text{X}}_{\left(4\right)}^{2}$ = 35.6, *p* < 0.001]. At Dwass-Steel-Critchlow-Fligner pairwise comparisons, participants who used the Internet “much more than before” COVID-19 pandemic showed statistically higher CSS-12 total scores than participants who used the Internet “much less” than before the pandemic (W = 5.47, *p* < 0.001), “less than before” (W = 5.87, *p* < 0.001) or “as before” (W = 7.03, *p* < 0.001).

Furthermore, most of the responders browsed the web for COVID-19 related information on a weekly basis (46.3%) and 15.9% daily. Lastly, 42.8% of participants reported finding a variable degree of struggle in resisting the search or in stopping during searches for COVID-19 related information. However, CYB symptoms severity did not differ considering these variables. Similarity, CYB emerged not to be influenced by the degree of trustworthiness participants have about COVID-19 related information found online.

### Cyberchondria Correlation With Psychopathological Factors

Table 4 shows the correlation between CYB and the psychopathological factors investigated.

**Table 4:** Correlations between the Cyberchondria Severity Scale (CSS-12) and psychometric questionnaires.

	**CSS-12**	**CAS**	**HADS-A**	**SHAI**	**HADS-D**	**RSES**	**IAT**	**MCQ-HA**	**WHOQOL**	**MOCQR**
CSS-12	–	0.323^**^	0.380^**^	0.560^**^	0.293^**^	−0.120^**^	0.332^**^	0.450^**^	−0.158^**^	0.339^**^
		*p* < 0.001	*p* < 0.001	*p* < 0.001	*p* < 0.001	*p* =0.004	*p* < 0.001	*p* < 0.001	*p* < 0.001	*p* < 0.001
CAS		–	0.464^**^	0.345^**^	0.324^**^	−0.189^**^	0.179^**^	0.261^**^	−0.172^**^	0.217^**^
			*p* < 0.001	*p* < 0.001	*p* < 0.001	*p* < 0.001	*p* < 0.001	*p* < 0.001	*p* < 0.001	*p* < 0.001
HADS-A			–	0.525^**^	0.644^**^	−0.490^**^	0.360^**^	0.349^**^	−0.468^**^	0.484^**^
				*p* < 0.001	*p* < 0.001	*p* < 0.001	*p* < 0.001	*p* < 0.001	*p* < 0.001	*p* < 0.001
SHAI				–	0.424^**^	−0.268^**^	0.366^**^	0.452^**^	−0.272^**^	0.416^**^
					*p* < 0.001	*p* < 0.001	*p* < 0.001	*p* < 0.001	*p* < 0.001	*p* < 0.001
HADS-D					–	−0.499^**^	0.371^**^	0.306^**^	−0.559^**^	0.373^**^
						*p* < 0.001	*p* < 0.001	*p* < 0.001	*p* < 0.001	*p* < 0.001
RSES						–	−0.348^**^	−0.113^**^	0.501^**^	−0.353^**^
							*p* < 0.001	*p* = 0.007	*p* < 0.001	*p* < 0.001
IAT							–	0.265^**^	−0.271^**^	0.334^**^
								*p* < 0.001	*p* < 0.001	*p* < 0.001
MCQ-HA								–	−0.236^**^	0.362^**^
									*p* < 0.001	*p* < 0.001
WHOQOL									–	−0.315^**^
										*p* < 0.001
MOCQR										–

*CAS, Coronavirus Anxiety Scale; CSS-12, Cyberchondria Severity Scale; HADS, Hospital Anxiety and Depression Scale; SHAI, Short Health Anxiety Inventory; RSES, Rosenberg's Self-Esteem Scale; IAT, Internet Addiction Test; MOCQR, Maudsley Obsessional-Compulsive Questionnaire; MCQHA, Meta-Cognitions about Health Questionnaire; WHOQOL, WHO Quality of Life-BREF. Spearman's r was used for correlations. Significant correlation*,

** *p < 0.05*.

CYB seemed to be related to different phenomenologies of anxiety, as the CSS-12 positively and strongly correlated with health anxiety (SHAI: *r* = 0.56, *p* < 0.001) and moderately correlated with general anxiety (HADS-A: *r* = 0.36, *p* < 0.001), anxiety specific to COVID-19 (CAS: *r* = 0.32, *p* < 0.001), and metacognitive health anxiety believes (MCQHA: *r* = 0.45, *p* < 0.001). Moreover, CYB emerged to be moderately correlated with higher expression of obsessive-compulsive symptoms (MOCQR: *r* = 0.34, *p* < 0.001), problematic usage of the Internet (IAT: *r* = 0.33; *p* < 0.001), and depressive symptoms (HADS-D: *r* = 0.38, *p* < 0.001). Conversely, a better quality of life and higher self-esteem emerged as possible protective factors for CYB, as shown by negative, though weak, correlations (WHOQOL-BREF: *r* = −0.16; *p* < 0.001; RSES: *r* = −0.12, *p* = 0.002).

## Discussion

The present study was aimed to describe behavioral Internet browsing routines for health-related information and CYB during the third wave of COVID-19 pandemic in an Italian large sample of the general population, exploring demographics and psychopathological factors most frequently related to this new potentially compulsive addictive digital behavior. Results showed that CYB exhibited a greater symptoms expression in females, younger individuals, students, and in subjects with physical or psychiatric conditions. Furthermore, psychopathological factors seem to play a role in the pandemic era regarding the positive correlation between CYB and health anxiety, COVID-19 related anxiety, metacognitive beliefs on health anxiety, obsessive-compulsive symptoms, and problematic usage of the Internet.

First, our findings showed that the Internet has been the most common source for health-related information searching during the pandemic outbreak, as it used to be during the pre-pandemic period as well (55). In our sample, 36.9% of the subjects used the Internet for this purpose and the frequency of online health searches increased in one out of three participants. In an epidemiological survey conducted in Italy in 2019 by the statistical office of the European Union, 35% of individuals searched for medical information online, a percentage that increased up to 46% during 2020 (56). Possible reasons underlying this trend can be: in general, the availability and easy accessibility of the Internet; specifically during the COVID-19 pandemic, the social isolation and the related heightened perception of health threats resulting in raising anxiety and distress that eventually lead to health-related queries. In more vulnerable individuals, searching on the Internet for health-related information might be compulsive, repetitive, hard to resist, and associated with negative consequences, depicting the pathological behavior of CYB. Although a defined cut-off of CYB has not yet defined in the literature, in the present sample more than 40% of subjects have reported they found it hard to resist or to stop using the Internet to search for COVID-19 health-related information. Moreover, our results have shown that subjects who increased the frequency of searching for health-related information online (compared to the time before the COVID-19 outbreak) and subjects who relate to the Internet as main source of information (compared with other more reliable sources) report higher CYB symptoms severity. These results could be related to the large amount of misleading, ambiguous, and inaccurate information accessible online (particularly in pandemic time) and to the unfitness of the Internet give reassurance, prompting people to continuing web-searching in excessive and compulsive way (24, 34, 57).

To assess sociodemographic and psychopathological factors more frequently related to CYB, this study investigated different independent variables. More severe CYB symptoms have been shown in female subjects compared to males. This outcome is in accordance with some previous investigations of pandemic time: most of those studies (22, 38, 58), but not all (40, 59), showed a positive correlation between female gender and the possible arising of CYB. Investigations conducted before the pandemic had found no connection between gender and CYB (32, 33). Women are generally more at risk to develop anxiety disorders than men (60) and several investigations on the psychological and mental-health impact of COVID-19 observed a heavier mental burden in females and increased depressive and anxiety symptoms (9, 61, 62).

With respect to the age, younger subjects showed higher CYB symptoms severity. This outcome has been corroborated in previous investigations conducted during the COVID-19 pandemic, that showed how older people are less affected by CYB. This can likely be related to the exposure of the seniors to less information overload and reduced sharing of junk news (22, 58). Higher CYB score in younger participants might also simply reflect the digital divide: one of the reasons why students have shown most severe CYB symptoms compared to retired subjects. Additionally, students and younger subjects more frequently use social media platforms which have been associated with a heightened risk of misinformation and CYB during the pandemic era (40, 43).

Considering COVID-19 related variables, CYB symptoms severity seem not depending on getting tested for SARS-CoV-2 (subject or relatives) nor on the loss of a dear one due to SARS-CoV-2. To the best of our knowledge, the severity level of CYB in those who have been directly in contact with the virus has not been previously reported. According to the most recent literature data, the exposure to the COVID-19 infection does not automatically express a negative psychological impact, with some investigations showing more severe depressive and anxiety symptoms in infected subjects (9, 63), while other studies did not support this association (64). The response to a stress/traumatic event is not univocal, while personality traits and psychological factors mediating this response (65). This might be true for CYB as well: the exposure to a stress/traumatic health-event might not directly and uniquely affect the behaviors related to the access and use of the Internet and the CYB itself, and possible further intervening factors might need deeper investigation in future studies. We could hypothesize that direct contact with the virus might lead the subjects to focus the attention to the stressor/traumatic event, leaving less mental space for other thoughts, such as worries, or the need of genuine reassurance about their own personal health.

Additionally, the presence of a physical or psychiatric disease was found to be associated with higher CYB symptoms severity. A previous investigation conducted during the pandemic era confirmed this association, showing higher CYB symptoms severity in patients with psychiatric disorders (i.e., Major Depressive Disorder, Anxiety Disorders, and Obsessive-Compulsive Disorders) compared to healthy controls (59). In this respect, the presence of underlying anxiety, depression, or obsessive-compulsive symptoms might be a predisposing factor for excessive and repetitive searches for health-related information and for CYB.

The reasons underpinning the higher CYB symptoms severity observed in subjects with a physical health condition can only be speculated, since no previous study conducted during the COVID-19 pandemic investigated this association. Already before the pandemic time, studies have shown that subjects coping with chronic disease largely indulge in surfing the web in order to search for health-related information, probably to find relief and help in managing the chronic conditions (66, 67).

Chronic patients are at high risk of severe consequences and death because of COVID-19 (68), and they might perceive it as a great surviving threat which in turn might fuel health anxiety and consequently CYB. Lastly, because of their deep need of reassurance and related behaviors (69), chronic patients are likely to be prone to frequent web surfing and eventually increasing CYB.

In the present study, the correlation between CYB and pre-existing psychopathological factors (already correlated with CYB in the pre-pandemic era) was confirmed. CYB positively correlated with problematic usage of the Internet which has been reported as one of the stronger predictors of CYB (28). In the pandemic era Internet-based addictive behaviors have increased (70) and some subject can express different rate of dysfunctional Internet-related behaviors, addiction, and impulsiveness that in turn might favor the onset or deepening of CYB. Moreover, our study confirmed the positive and strong correlation between CYB and health anxiety and the moderate correlation with general anxiety and anxiety about COVID-19. These outcomes are in accordance with previous literature findings, claiming the fear of COVID-19 positively correlated with CYB (35–37). Additionally, the present study extended the knowledge of the impact of health-related metacognitive believes (positively correlated with CYB) confirming the role of metacognition (e.g., “Worrying about my health will help me to cope”) as factors related to higher expression of CYB (32) and showed a positive correlation between CYB and obsessive-compulsive symptoms. No previous investigation analyzed this correlation during the COVID-19 pandemic, but an exacerbation of obsessive-compulsive symptoms has been reported during the pandemic (71) and in patients with Obsessive-Compulsive Disorder (72). Considering CYB as a potentially new form of compulsive digital behavior (25), the COVID-19 pandemic is likely to have had an impact on CYB mediated by obsessive-compulsive behaviors.

The present findings showed that CYB positively correlates with depressive symptoms as well, while this correlation was reported to be minimal in the pre-pandemic era (28, 73).

Lastly, CYB negatively correlated with self-esteem and quality of life, validating previous evidence between low self-esteem and higher CYB symptoms severity (33). Subjects with low self-esteem might prefer to avoid face-to-face healthcare, and because low self-esteem has been associated with problematic Internet use (74), again two characteristics of this pandemic era.

The correlation between CYB and poor quality of life has not enough been investigated until today. To the best of our knowledge, only one previous study conducted before the COVID-19 pandemic reported CYB was uniquely associated with functional impairment, not with the quality of life (23). Our results showed the negative correlation between quality of life and CYB. We can assume that participants with lower perception of health and well-being might be more vulnerable to repetitive online searches and CYB or, conversely, that CYB might have negative consequences and implications for the quality of life, thus highlighting the need for monitoring and clinical intervention.

## Study Limitations

The present results must be interpreted in light of some limitations. First, data have been collected by an online survey: enrolled subjects are likely to belong to a specific part of the general population, with easy Internet access, familial and confident in the web as a source of information, then likely to manifest higher CYB symptoms severity. Online surveys also have potential methodological biases (e.g., genuineness of the responses) which have not been addressed in the present study. Additionally, all variables were self-reported, hence liable to be affected by established method biases. The cross-sectional nature of the study temporally limited the recruitment to the period of the COVID-19 third wave in Italy and the results might change depending on the evolution of the outbreak. However, we gathered our data during a very special, thus dramatic period of time that allowed us to report the impact of several lockdowns on CYB and related variables, something that no other research has investigated so far. The sample mainly consisted of subjects living in Lombardy, therefore the outcome generalization to the whole national sample must be cautious. The cross-sectional design does not allow to demonstrate actual correlations and causality directions considering the study's findings. Therefore, future longitudinal studies are warranted in order to establish actual causality.

## Conclusion

Based on the present results, it can be concluded that CYB showed a greater symptoms expression in females, younger subjects, students, and in patients with physical or psychiatric illness. Psychopathological factors have been reported to play a role in the pandemic era, confirming that CYB was positively correlated with health anxiety, anxiety about COVID-19, metacognitive believes about health anxiety, obsessive-compulsive symptoms, and problematic usage of the Internet. On the contrary, high self-esteem and good quality of life were found to be protective factors for CYB symptomatology. Ultimately, the present investigation can help to understand how the trend of accessing the Internet to seek health-related information typically increases during public health emergencies, what specific psychopathological factors may be associated with CYB, and the need to set up effective intervention in clinical settings in order to prevent this compulsive new digital behavior.

The guidelines for prevention in psychology proposed by the American Psychiatric Association (75) underline that preventive interventions and programs should aim at reducing personal and environmental risk factors and, at the same time, increasing protective factors. Considering the results of the present study, preventing and/or reducing CYB interventions can target the most vulnerable subjects, like young people in settings like school, focusing on improving awareness by psycho-education strategies and teaching more functional relationships with the web, how to reduce the time spent online and increasing awareness on positive and negative factors of this platform as well as possible risks of developing pathological behaviors (76). Our time and the current outbreak push for the increasing need for health-related information, public health interventions through policymaking might also be of value [e.g., provision of more precise, user-friendly, and unambiguous medical information, institutional control on the spread of “fake news” and misinformation, improving official and government health websites (25)]. In clinical settings, professional evaluation should include identifying the presence of psychopathological factors that might promote a maladaptive use of the Internet. As observed in our study, specific attention should be paid to subjects who show higher levels of health anxiety, fears of COVID-19, obsessive-compulsive symptoms, and problematic usage of the Internet. These subjects might be considered at higher risk of developing CYB and might benefit from specific interventions. Thus far, only one randomized controlled trial showed Internet-delivered Cognitive Behavioral Therapy to be effective in reducing CYB in patients with a DSM-5 diagnosis of Illness Anxiety Disorder and/or Somatic Symptom Disorder (77); this improvement was mediated by decreased health anxiety, a variable strongly correlated with CYB. Lastly, promoting psychological interventions that showed to improve self-esteem (78) might be of value in reducing CYB.

## Data Availability Statement

The raw data supporting the conclusions of this article will be made available by the authors, without undue reservation.

## Ethics Statement

The studies involving human participants were reviewed and approved by University of Milan Ethics Committee (number 27/21). Written informed consent for participation was not required for this study in accordance with the national legislation and the institutional requirements.

## Author Contributions

All authors were involved in drafting the manuscript, agreed to its publication, and read and approved the final version of the manuscript.

## Conflict of Interest

The authors declare that the research was conducted in the absence of any commercial or financial relationships that could be construed as a potential conflict of interest.

## Publisher's Note

All claims expressed in this article are solely those of the authors and do not necessarily represent those of their affiliated organizations, or those of the publisher, the editors and the reviewers. Any product that may be evaluated in this article, or claim that may be made by its manufacturer, is not guaranteed or endorsed by the publisher.

## Appendix

Questions included in the online survey. Q: question; A: answers. Q: How much time do you spend on the Internet on average every day (for work/study/leisure/other reasons)? A: <1 h; 1–3 h; 3–5 h; > 5 h.

Q: Which is the source you use the most to research information about your health? A: Health professional/general practitioner; Internet; scientific journals/encyclopedias etc.; other; TV/radio; non-professional information by friends and relatives.

Q: Since the COVID-19 outbreak, how often do you use the Internet to search for health-related information? A: much less than before; less than before; as before; more than before; much more than before.

Q: How often do you search for COVID-19 health-related related information? A: less than once a week; once a week; every 3/4 days; every day; several times a day.

Q: Did you find hard to resist or to stop searching for health-related information about COVID-19? A: never; occasionally; sometimes; often; always.

Q: Do you believe in online health information about COVID-19 without verifying the source and the validity of these information?

A: never; occasionally; sometimes; often; always.

Q: Did you test positive for SARS-CoV-2? A: yes/no

Q: Did someone among your relatives test positive for SARS-CoV-2? A: yes/no

Q: Did you loss a dear one because of COVID-19? A: yes/no
